# Submental island flap reconstruction reduces cost in oral cancer reconstruction compared to radial forearm free flap reconstruction: a case series and cost analysis

**DOI:** 10.1186/s40463-016-0124-8

**Published:** 2016-02-05

**Authors:** D. Forner, T. Phillips, M. Rigby, R. Hart, M. Taylor, J. Trites

**Affiliations:** Division of Otolaryngology – Head and Neck Surgery, Department of Surgery, Dalhousie University, Halifax, Canada

**Keywords:** Oral cancer, Reconstruction, Submental Island Flap, Radial Forearm Free Flap, Cost, Pedicle Flap, Free Flap

## Abstract

**Background:**

In Canada, 4,400 cases of oral cancer are diagnosed yearly. Surgical resection is a key component of treatment in many of these cancers. Reconstruction of defects, with the goal of preserving function, is of utmost importance. Several choices are possible for reconstruction of larger defects, including both free and pedicled flaps. Free flap reconstruction is reliable and effective, but requires additional personnel and peri-operative resources. Pedicled flaps remain an important alternative to free flaps, and are less resource intensive. This paper reviews our inaugural experience with the submental island flap (SIF) and compares costs incurred to a matched cohort of oral cancer patients reconstructed with forearm free flaps.

**Methods:**

Charts of patients who underwent SIF and RFFF reconstruction from January 1^st^ 2013 to April 1^st^ 2015 were retrospectively examined. Associated costs were obtained via online database and previously reported costs at the study institution.

**Results:**

Mean length of ICU stay in glossectomy RFFF reconstruction was 4.7 days. Only one patient required ICU stay for one night in the SIF group. Mean length of hospital stay was not significantly different in SIF patients vs RFFF patients (12.4 vs 15.4 days, p > 0.05). Mean operative time was significantly lower in the SIF group compared to the RFFF group (347 vs 552 min, p < 0.05). Total mean intraoperative costs were found to be $4780.59 for RFFF operations, versus $2307.94 for SIF. Total mean cost of post-operative stay was $18158.40 in the SIF group and $43617.60 in the RFFF group. Total cost savings were therefore $27931.85 per patient for the SIF group.

**Conclusions:**

We have demonstrated the use of the submental island flap as an alternative to radial forearm free flaps, showing both decreased hospital costs and comparable patient outcomes. Pedicled flaps are making a resurgence in head and neck reconstruction, and the submental island flap offers an excellent alternative to more labour intensive and costly free flap alternatives.

## Background

Head and neck cancer accounts for over 500,000 cancer diagnoses worldwide [1], with approximately 3 % of new cancer cases in the United States being head and neck in origin [2]. In Canada, 4400 new cases of oral cancer are diagnosed yearly [3]. Surgical resection is typically a key component of treatment in most cancers originating in the oral cavity, pharynx, face, or neck [4]. For larger mucosal defects, primary reconstruction is often pursued to optimize functional outcomes. In addition to its social and esthetic importance, the head and neck region is also fundamental in speech, swallowing, and respiration. Consequently, reconstructive options in head and neck surgery have been studied extensively. Tissue flaps are one such option, and these include both free flaps and pedicled flaps [5]. Free tissue transfer has become a mainstay of reconstruction in recent years, offering improved vascularity and wound healing, potential for innervation, tailoring of the wound defect, and a wide variety of tissue options [5]. These flaps have become the standard against which other means of reconstruction must be evaluated.

However, free flap reconstruction is limited due to increased operative time, and need for specialized equipment and microvascular expertise [6]. A lengthy hospital admission can also be anticipated. In a context of limited health care resources, the indiscriminate use of free tissue transfer allows fewer patients to receive timely surgical care. Not surprisingly, free flap operations have been associated with greater costs in both the intraoperative and immediate post-operative periods [7].

Health care costs in Canada have increased every year since 1975. Between 2000 and 2010, health care costs increased an average of 7 % per year. In the past four years, health care spending has continued to rise, albeit at a slightly slower rate [8]. Together with a modest but steady climb (1.2 %/yr) in the incidence of oral cancer in Canada [9], some of which is likely attributable to human papillomavirus, these realities demand a revisiting of more economical alternatives for reconstruction in head and neck surgery.

Quite independent of these fiscal pressures, pedicled flaps have re-emerged as important alternatives to free flaps. In particular, the submental island flap has recently grown in popularity. Originally reported in 1993 by Martin et al. [10], the submental island flap is a fasciocutaneous flap derived from the submental region, and is supplied by the submental vessels of the facial artery [11]. When mobilized on its vascular pedicle, the flap exhibits great flexibility and can be transposed into a number of locations. The flap is commonly employed in oral reconstruction (tongue, floor-of-mouth, buccal vestibule, palate), but other indications include defects of the oropharynx, hypopharynx and lower face.

The submental island flap offers several advantages. Like all pedicled flaps, it obviates the need for microvascular surgery. This feature alone would be expected to reduce operative time. We hypothesized that it would also reduce the demand on specialized care (ICU) as well as total hospital stay, and, consequently, reduce the overall costs associated with surgical care. Although it is beyond the scope of this paper, this flap has proven to be exceptionally reliable, and to provide plenty of pliable soft tissue up to 75 cm^2^. The donor site is largely obscured by the chin, and older patients or those with significant skin redundancy (jowling) can enjoy a sharpened cervico-mental angle following the procedure. Although some surgeons have been reluctant to embrace the SIF on oncologic grounds, we have found it to afford a comprehensive level I lymphadenectomy, and this has been supported by high volume longitudinal studies [12].

These advantages make the SIF a good option for selected surgical defects which might otherwise have been reconstructed with free flaps. As with all reconstructive modalities, these pedicled flaps are not without potential disadvantages. In the SIF, these include excess tissue bulk and hair (beard)-bearing skin. As with the forearm free flap, these issues may need to be addressed with additional procedures. In most cases, neither bulk nor hair are issues if radiation therapy is required in the adjuvant setting.

In this paper we review a series of submental island flaps used for a broad range of indications. Recognizing the limitations of doing so in a public health system, we also estimate costs associated with this procedure, and compare these with a similar group reconstructed with forearm free flaps. In order to ensure homogeneity among the two groups, enrolment was restricted to patients receiving nearly identical oncologic surgery, including partial (up to 50 %) glossectomy with ipsilateral selective neck dissection. All patients also received a temporary tracheostomy. Costs are compared between those patients whose surgical defects were reconstructed with SIFs and those whose defects were reconstructed with forearm free flaps. We hypothesized that the SIF would offer substantial cost savings when compared to RFFF operations.

## Methods

### Patient demographics and outcomes

For initial assessment, all patients who received submental island flap reconstruction from January 1^st^ 2013 to April 1^st^ 2015 were examined retrospectively. All patients who received radial forearm free flaps over the same time period were evaluated as potential comparators. Within each of these two groups, those patients who met the inclusion criteria (surgery limited to tracheotomy, <50 % glossectomy, and ipsilateral neck dissection) were included for the comparison. Cancer staging was based on the sixth edition of the tumor-node-metastasis staging system for head and neck cancer by the American Cancer Society [13]. Comorbidity scoring was based on the American Society of Anesthesiology (ASA) risk stratification score.

### Cost analysis

Cost analysis was based on a cost difference method, where modalities similar in both SIF and RFFF reconstruction were effectively negated between the groups. This included pre-operative workup and consultation, intra-operative and post-operative pathology and pathologist costs, post-operative follow-up, and adjuvant therapy. Therefore, only costs associated with the operative procedure and post-operative hospital stay were included in the analysis. This included anesthesia costs, nursing costs, surgeon costs, operative consumable costs, ICU costs, and hospital stay costs. Additionally, an alternative cost difference analysis was completed using an estimation of the cost associated with flap debulking following SIF reconstruction.

Nursing costs were obtained by averaging the minimum and maximum hourly wages, as obtained by www.careerbeacon.com. This average was then included in calculations involving procedure time and number of nurses required for the procedure. Costs associated for remaining areas was obtained by contacting the Queen Elizabeth II Health Science Centre Business Department. Surgeon and anesthesiologist salaries were obtained from a previous publication in the division of otolaryngology – head and neck surgery at our institution [14]. Briefly, billing codes were used to determine the annual salary of Head and Neck Surgeons, which was divided by the average number of hours worked per week to yield an average hourly wage.

### Statistical analysis and research approval

Statistical analysis was completed using the commercially available software SPSS (v21; IBM, Chicago, Illinois). Categorical variables were compared using either Chi-square test with or without Monte Carlo procedure (iteration = 10,000 cross tables). Continuous variables were compared using Student’s *T*-Test or Mann–Whitney *U*-Test.

The Nova Scotia Research Ethics Board has approved this study as a Quality Assurance/Delivery of Care Initiative under Article 2.5 of the Tri-Council Policy Statement 2.

## Results

### Submental island flap reconstruction series patient demographics

A total of 12 patients were identified within the study period that were scheduled for submental island flap reconstruction. Two of the 12 were converted during the perioperative period to supraclavicular flap reconstruction, and one was converted to primary defect closure. Therefore, nine patients remained who underwent submental island flap reconstruction. All of the procedures were performed by a single surgeon (JT). Follow-up time ranged from 8 days to 746 days (Table 1; mean 272 days).

**Table 1:** Submental island flap group details

Patient	Age	Sex	Defect	Pathology	Flap size	Complications
1	53	F	Anterior tongue to base of tongue	Carcinoma in situ	7 x 4	Revision (tethering)
2	80	F	Retromolar trigone, palate, and oropharynx	T4 N0	14 x 6	
3	57	F	Left anterior tongue	pT2 N1 M0 SCC	8 x 5	Debulking, flap dehiscence
4	84	F	Maxilla, Hard palate, retromolar trigone	T4 N0 SCC		
5	68	M	Anterior and mid tongue, floor of mouth	T2 N0 SCC	12 x 4.5	Debulking
6	50	M	Tongue Base, floor of mouth	T2 N0		Depilation, debulking
7	60	F	Osteoradionecrosis of right mandible	N/A		External flap failure
8	74	M	Tongue, oropharynx	T3 N2a	14 x 5	
9	59	M	Tongue, floor of mouth		13 x 4.5	

There was no strong gender predominance (44 % male), and the mean age at time of procedure was 65 years of age. Tumor size was generally large, with 33 % of patients having T3 or greater primary tumor size, and no tumor involvement less than T2. Defects commonly involved the tongue (66 %), floor of mouth (33 %), or palate (22 %). Full defect involvements for all patients are listed in Table 1.

### Submental island flap reconstruction series patient outcomes

Flap sizes ranged from 7x4cm to 14x6cm, with a mean area of 37.4 cm (Table 1). All donor sites were closed primarily by local tissue advancements. The most common complications were requirement for debulking (33 %) and depilation (11 %). One patient required revision for reasons other than debulking (sulcus reconstruction). One patient experienced flap failure. This is believed to be the result of draping of the vascular pedicle over a heavy reconstruction plate.

Mean length of hospital stay in submental island flap reconstruction patients for all indications was 12.4 days, with a mean operative time of 346 min.

### Submental island flap vs radial forearm flap glossectomy reconstruction comparison

There were nine patients in the SIF glossectomy group, and 12 patients in the RFFF group. The SIF group and the radial forearm free flap (RFFF) did not differ significantly in gender distribution, age, stage distribution, or comorbidity score (*p* > 0.05, Table 2). The RFFF did have significantly larger flap areas (56.9 vs 69.0 cm^2^, *p* < 0.05, Table 2). No patients in the RFFF group required debulking or depilation (Table 3). Complications in the RFFF group are outlined in Table 3.

**Table 2:** Comparison of gender, age, tumor staging, and flap size

Variable	SIF	RFFF	Statistic
Gender (%)	43	75	*p* > 0.05
Age (years)	63	65	*p* > 0.05
Stage^a^			*p* > 0.05
ASA	2.4	2.3	*P* > 0.05
Flap Area	56.9	69.9	*p* < 0.05

^a^TNM is tumor (range T2 – T4), node involvement (range N0 – N2c), and distant metastasis (none in this study)

There was no significant difference in gender, age, or tumor stage

**Table 3:** Radial forearm free flap group details

Patient	Age	Sex	Defect	Pathology	Flap size	Complications
A	55	F	Left lateral tongue, pharynx, floor of mouth	T3N2c SCC	10 x 7	
B	64	M	Left tongue, right pharynx	T3N2b SCC	12 x 10	
C	61	M	Right tongue, right pharynx, right floor of mouth	T3N0 SCC	9 x 6	
D	71	M	Left tongue, left floor of mouth	T2N0M0 SCC	9 x 6	
E	63	M	Tongue, pharynx	T1N0M0 SC	12 x 6	
F	66	M	Tongue, oropharynx	T4aN0M0 SCC	10 x 12	
G	55	M	Tongue, floor of mouth	T3N0M0 ACC	8 x 5	Revision (tracheostomy teathering)
H	55	F	Left tongue, left pharynx, left floor of mouth	T3N2c SCC	10 x 7	
I	80	F	Left tongue, left floor of mouth	T2N0 SCC	8 x 5	
J	64	M	Left tongue, floor of mouth	T2N1 SCC	6 x 5	Hematoma (neck, left forearm), dysphagia, forearm wound infection
K	73	M	Right tongue	T2N1M0 SCC	8 x 4	
L	69	M	Tongue, pharynx, mandible	T4aN1	14 x 9	

**Fig. 1:**
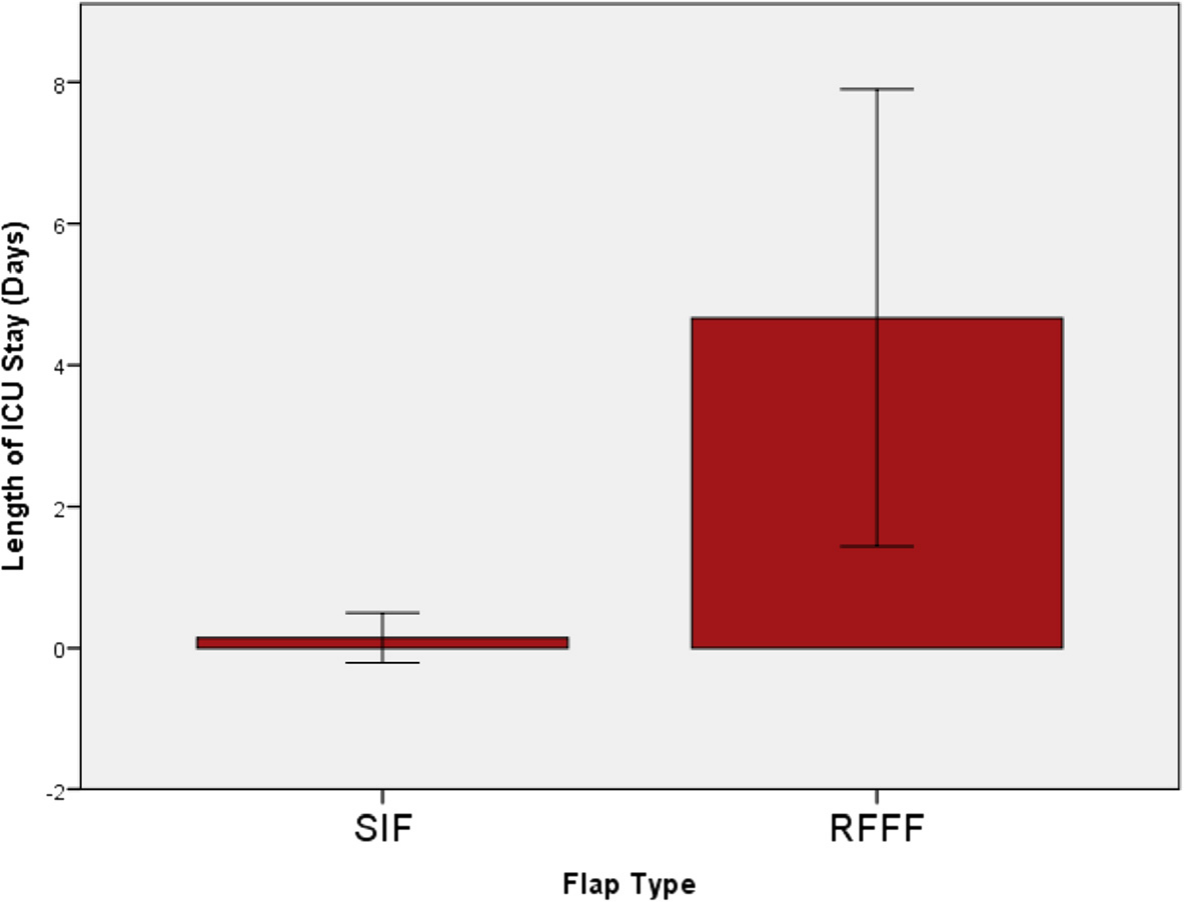
Mean length of ICU stay was 4.7 days in the RFFF group. Only one patient required ICU stay for a single night in the SIF group

**Fig. 2:**
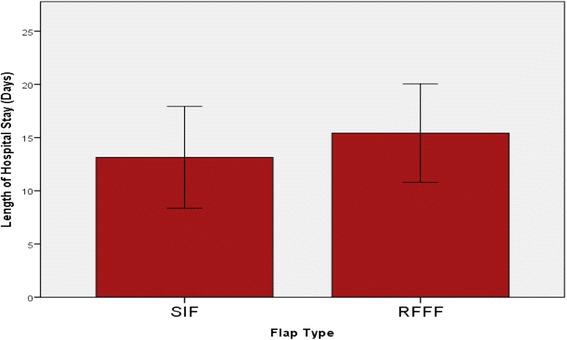
The mean length of hospital stay was not significantly different between SIF patients and RFFF patients

**Fig. 3:**
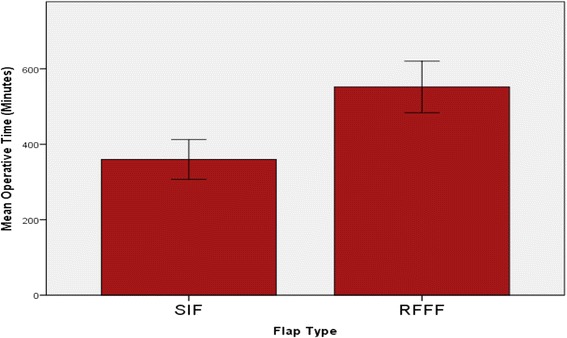
The mean operative time was significantly lower in the SIF group as compared to the RFFF group

Mean length of ICU stay in glossectomy reconstruction RFFF patients was 4.7 days (Fig. 1). Only one patient required ICU stay for one night in the SIF group, giving a mean length of ICU stay of 0.14 days (Fig. 1). Mean length of hospital stay was not significantly different in SIF patients vs RFFF patients (12.4 vs 15.4 days, p > 0.05, Fig. 2). Mean operative time was significantly lower in the SIF group compared to the RFFF group (347 vs 552 min, *p* < 0.05, Fig. 3).

### Cost analysis

For this study, mean hourly wage was calculated at $44.72 for nurses, $125 for anesthesiology and $140 for surgeons. One additional nurse is required for six hours in RFFF operations. Similarly, each RFFF operation requires two surgeons. The cost of the extra surgeon was estimated using the time required of the additional nurse.

Intraoperative costs are summarized in Table 4. Using the above salaries, nursing costs for RFFF operations were 1.9 times greater than the nursing costs for SIF operations, anesthesiologist costs were 1.6 times greater, and surgeon costs for RFFF operations were 3.2 times greater. Total mean intraoperative costs were found to be $4780.59 for RFFF operations, versus $2307.94, yielding a total cost difference of $2472.65. This represents a cost increase of 2.1 times in the RFFF group. The greatest contributor to intraoperative cost savings were the costs of an additional surgeon for an extended operation length, resulting in a difference of $840.00.

**Table 4:** Intraoperative cost breakdown

Flap Type	HCP	Number of HCP	Hourly wage ($/h)	Hours	Total Cost ($)
SIF	Nurse	3	44.72	5.78	775.45
	Surgeon	1	140	5.78	809.62
	Anaesthesiologist	1	125	5.78	722.88
RFFF	Nurse	3	44.72	9.2	1234.27
	Extra Nurse	1	44.72	6	268.32
	Surgeon	1	140	9.2	1288.00
	Extra Surgeon	1	140	6	840.00
	Anaesthesiologist	1	125	9.2	1150.00

HCP = Health care professional, SIF = Submental island flap, RFFF = Radial forearm free flap

One night in hospital was found to be $1404.00 per night, while one night in an ICU bed was calculated to be $6084.00 per night. This equated to a total mean cost of $18158.40 in the SIF group and $43617.60 in the RFFF group, yielding a cost difference of $25,459.20. This represents a cost increase of 2.4 times in the RFFF group for post-operative hospital stay alone, primarily due to the cost of ICU beds. Combining cost differences in both the intraoperative setting and postoperative hospital stay yields a total cost savings of $27931.85 for the SIF group. The greatest contributor to this total difference was the cost of post-operative stay (90.2 %).

The most common post-operative revision required in the SIF group was flap debulking. This is completed in the minor procedure clinic, and requires one nurse and one surgeon for one hour. Analysis shows an estimated cost of $184.72 for this procedure.

## Discussion

With rising health care costs in Canada, concerns over the high costs of surgical care is well justified. Historically, the use of pedicled flaps in head and neck reconstruction has been overshadowed by the use of free flaps. However, free flaps are typically associated with longer operative times and increased length of hospital stays. Furthermore, free flaps have a requirement of intensive care monitoring in most centers for part of the post operative period. Due to these factors free flaps are associated with a much higher cost to the medical system and a more cost efficient option should be considered.

In direct comparison to radial forearm free flap reconstruction, submental island flap reconstruction was associated with shorter operative times and length of hospital stay. Furthermore, only a single patient in the SIF group required any intensive care monitoring, and in total spent a single day in the ICU. This is opposed

to the RFFF group that required a minimum of one night ICU stay, and had a mean length of ICU stay much greater than this. It is institutional policy that all patients receiving any free flap spend a minimum of one night under intensive care monitoring.

The main objective of this study was to demonstrate the cost effectiveness of submental island flap reconstruction. It should also be noted, however, that SIF reconstruction does indeed offer excellent functional and cosmetic outcomes, particularly at the donor site. We have demonstrated the use of submental island flaps as a reliable and safe procedure in many forms of head and neck cancer reconstruction. Patients in our case series had very few complications, the majority of which were non life threatening. This is in keeping with previous studies which have shown the low morbidity and mortality rates of submental island flap reconstruction [7, 15–18]. Furthermore, when compared to RFFF reconstruction patients, SIF reconstruction patients had a similar incidence of complications, also with low severity.

Common disadvantages of SIF reconstruction often cited are the need for depilation and potential for recurrence due to submental and submandibular nodal involvement. Hair growth may be an issue in patients with thick, fast growing facial hair. In patients requiring adjuvant radiotherapy, hair growth quickly ceases. Recent studies have begun examining the most effective forms of depilation in SIF reconstruction patients, with electrolysis and laser therapy typically showing preferred results [19]. Finally, should patients not receive radiotherapy and elect to not receive depilation therapy, it has commonly been noted that over time, hair growth in the flap ceases independently (mucosalization) [20]. In terms of recurrence risk, several studies have shown no increased risk of recurrence when there is no obvious nodal involvement [12]. This risk is further decreased with judicious node dissection and thinning of the flap during harvesting. Indeed, we have not yet experienced any recurrence when utilizing the submental island flap for reconstruction of head and neck cancers.

Another possible sequelae of SIF reconstruction is the need for flap debulking in the post-operative period. In this study, 33 % of patients required debulking of their flap. Disposables are also an associated cost of this procedure, but vary according to the patient and specific procedure, and are generally low. We have therefore estimated the cost of flap debulking using only the associated personnel cost, and found it to be minimal when it is necessary.

The submental island flap also offers excellent cosmetic outcomes. The incision for flap harvesting may be hidden in the submental crease, behind the mandibular arch, and due to the nature of the harvest, many patients express enjoyment of their “neck tightening” procedure. Furthermore, when used for facial reconstruction, the color matching ability of the submental island flap is essentially unparalleled.

Further advantages of the submental island flap are seen with surgeon preferences, in that the submental island flap is a relatively easy and safe dissection. The technique of harvesting the flap has recently been modified to be safe for residents in training [21], potentially offering further widespread use. Tissue from the submental region is also thin, supple, and pliable, giving it ideal characteristics for reconstruction.

Our SIF group findings are comparible in terms of mean flap area and length of hospital stay to a previous study be Paydarfar et al. [17] examining SIF vs RFFF in oral reconstruction. Patients in our study experienced shorter operative times than this previous study, regardless of whether examining the glossectomy subset or full patient population.

The cost differences in this study were found to be in agreement with our hypothesis. Substantial cost differences were observed when comparing SIF reconstructions to RFFF reconstructions. This finding is similar to those reported by Miller et al. whereby submental island flaps were associated with a 40 % cost reduction in reconstruction of temporal bone defects as compared to free flaps [7].

Reduced cost associated with procedures is alluring in itself. However, in dividing the cost differences into their component parts, other advantages of SIF operations have been highlighted.

The majority of cost differences were due to ICU requirement following RFFF reconstruction. Utilizing pedicled flaps in order to reduce the number of patients requiring ICU beds would therefore increase ICU bed availability. Not all institutions include protocols for minimum length of ICU stay following free flap reconstructions. It is debated whether ICU monitoring is required in the post-operative period for free flap reconstruction [22]. However, some authors have supported a minimum ICU stay for patient safety and complication prevention [23]. For institutions in which post operative ICU stay is not required, the cost saving associated with pedicled flaps would be reduced. It is also important to note that institutions that do not require an ICU stay often still require a one-to-one nurse-to-patient ratio in the immediate post-operative period, which increases costs. Nonetheless, for institutions including post-operative ICU monitoring as part of their free flap protocols, pedicled flaps offer an opportunity to reduce cost and improve bed availability.

Despite contributing a lesser amount to overall cost savings, the cost differences and health care professional requirements are important. Free flap reconstruction in this study required an extra nurse for six hours, and an extra surgeon for those six hours. Pedicled flaps therefore offer the opportunity for hospital staff resources to be used more efficiently, and potentially allow additional procedures to be completed. Regardless of differences in cost, the increased time required for free flap reconstruction potentially covers the time of an additional pedicled flap operation (ie, three SIF operations may take place in the time required for two RFFF operations). Performing pedicled flaps when possible may therefore help reduce patient wait times by increasing the number of potential operations in a given time span. Furthermore, the decreased operative time associated with pedicled flap reconstruction may benefit patients unable to tolerate longer operative times. With relatively few downsides, pedicled flaps clearly offer many potential advantages over free flaps when the case is appropriate. Finally, pedicled flaps allow for treatment of time-sensitive cancer cases that would otherwise be post-poned or cancelled if ICU beds were not available.

There are limitations to this study. The sample population was small, although it does represent the majority of submental island flap reconstructions at this institution. The two groups used for comparison were not perfect matches. However, we were able to highlight the lack of statistical significance in gender, patient age, and tumor stage between SIF and RFFF groups. Flap area was larger in the RFFF group. This outlines that RFFF reconstruction may be more appropriate than SIF reconstruction when larger defects are expected. Limitations in the cost analysis portion of the study were also present. Namely, cost difference was completed as opposed to specific cost analysis. Several factors were assumed to be equivalent between RFFF and SIF operations, namely the preoperative work up costs and post-operative requirements unrelated to hospital stay, such as recurrence, follow-up visits, etc. However, we believe this is an accurate picture of the cost differences between pedicled and free flaps, and our analysis offers a valid preliminary study detailing the cost savings that are possible with pedicled flaps.

In summary, rising health care costs in Canada have called into question the utility of expensive surgeries when cheaper alternatives are available. We have demonstrated the use of the submental island flap as an alternative to radial forearm free flaps, showing both decreased hospital costs and adequate patient and cosmetic outcomes. Pedicled flaps are making a return to the field of head and neck reconstruction, and the submental island flap offers an excellent alternative to more intricate and demanding free flap alternatives

## Conclusions

Head and neck cancer remains a substantial contributor to total cancer incidence in Canada. Many of these cancers require reconstruction of surgical defects which vary in size. Rising health care costs dictate that more cost effective procedures should be considered where possible. Free flaps have become a staple in head and neck reconstruction, yet pedicled flaps offer shorter operative times as well as potential for faster patient recovery. In this study, we have detailed that the pedicled submental island flap offers adequate patient outcomes compared to the radial forearm free flap in glossectomy reconstruction, and offers substantial cost savings through reduced operative time and decreased length of ICU stay. This offers further support for the recent resurgence of pedicled flap use, specifically the submental island flap.

## Abbreviations

ICU: intensive care unit
RFFF: radial forearm free flap
SIF: submental island flap
SPD: shipping, processing, and delivery
TNM: tumor, node, metastasis (staging)
